# State of the Art in Rehabilitation Strategies After Hip Arthroscopy for Femoroacetabular Impingement Syndrome: A Systematic Review

**DOI:** 10.3390/jcm13237302

**Published:** 2024-11-30

**Authors:** Claudio Monselli, Luca Bianco Prevot, Riccardo Accetta, Livio Pietro Tronconi, Vittorio Bolcato, Giuseppe Basile

**Affiliations:** 1 IRCCS Ospedale Galeazzi—S. Ambrogio, Via Cristina Belgioioso 173, 20157 Milan, Italy; claudio.monselli@gmail.com (C.M.); riccacc@gmail.com (R.A.); basiletraumaforense@gmail.com (G.B.); 2 Residency Program in Orthopaedics and Traumatology, University of Milan, 20122 Milan, Italy; 3 GVM Care and Research, Maria Cecilia Hospital, 49033 Cotignola, Italy; ltronconi@gvmnet.it; 4 Department of Human Science, European University of Rome, 00163 Rome, Italy; 5 Astolfi Associates Legal Firm, 20122 Milan, Italy; bolcatovittorio@yahoo.it

**Keywords:** femoroacetabular impingement, rehabilitation, arthroscopic surgery

## Abstract

**Background/Objectives:** Femoroacetabular impingement (FAI) is a common cause of hip pain in athletes and active individuals, often requiring hip arthroscopy followed by a structured rehabilitation program. Effective rehabilitation is crucial for optimizing surgical outcomes and facilitating a return to sport. **Methods:** A systematic review following PRISMA guidelines was conducted to evaluate post-operative rehabilitation protocols after hip arthroscopy for FAI. Databases searched included PubMed, Embase, and Cochrane Library up to April 2024. Inclusion criteria focused on studies documenting rehabilitation post-arthroscopy for FAI, with a final selection of 14 studies encompassing 1105 patients. Data extraction focused on rehabilitation techniques, functional outcomes, and return-to-sport rates. The risk of bias was assessed using RoB2 and ROBINS-I tools. **Results:** Rehabilitation protocols were categorized into four phases, emphasizing manual therapy, strengthening, stretching, aquatic exercises, and sport-specific drills. The average return-to-sport rate was 90.3%. Manual therapy and aquatic programs were critical in early recovery, while strengthening and proprioception exercises were central to later stages. Variability in protocols across studies was noted. **Conclusions:** Post-operative rehabilitation following hip arthroscopy for FAI is essential for recovery. A phased, individualized approach, integrating manual therapy, aquatic exercises, and sport-specific training, yields positive outcomes. However, the heterogeneity of protocols suggests the need for standardized guidelines tailored to individual patient needs and activity levels.

## 1. Introduction

Femoroacetabular impingement (FAI) is a condition that characteristically presents with hip pain secondary to mechanical impingement from abnormal hip morphology involving the proximal femur and/or acetabulum. FAI is a leading cause of hip pain, particularly affecting young and athletic individuals, as well as those with high expectations of returning to sports and physical activity [1]. This condition is especially prevalent among athletes whose sports involve extreme hip movements, such as repetitive hyperflexion and rotational loading [2]. FAI can manifest in two primary forms: cam, characterized by abnormal morphology of the femoral head, and pincer, involving excessive acetabular coverage. Both types can contribute to early-onset osteoarthritis if left untreated [3,4]. While physical therapy can successfully manage symptoms in some cases, hip arthroscopy is often necessary for patients with more severe cases or higher physical demands [5].

Hip arthroscopy has become a common and effective treatment for FAI, with the number of procedures increasing dramatically over the past 25 years—by as much as 18-fold [6]. This rise reflects not only the growing recognition of FAI but also advancements in surgical techniques, which have resulted in fewer complications, quicker recovery times, and improved patient-reported outcomes [7]. Studies in the literature have shown that the surgical management of FAI leads to significant improvements in patient outcomes, with as many as 92% of athletes returning to their sport following arthroscopy [2,8].

However, despite the positive outcomes associated with surgical intervention, the crucial role of post-operative rehabilitation in achieving optimal results must be emphasized. Studies consistently show that patients who follow a structured physical therapy program after surgery experience superior outcomes, including enhanced range of motion and functional recovery, both in the short and long term [9]. It is clear that for patients to experience the best possible outcomes, surgery must be followed by an optimal rehabilitation program.

Despite the broad consensus on the importance of post-operative physiotherapy, there remains considerable variation in the rehabilitation protocols recommended in the literature. A four-phase post-surgical rehabilitation program is commonly cited as effective, but there is still a lack of uniformity in the specific exercises and timelines recommended during each phase [10]. This review aims to systematically examine and describe the various rehabilitation treatments available following hip arthroscopy for FAI, providing an overview of the current evidence and identifying the best practices to guide clinical decision making.

## 2. Materials and Methods

The review was conducted following the Preferred Reporting Items for Systematic Reviews and Meta-Analyses (PRISMA) guidelines [11]. The systematic review was registered and allocated in the PROSPERO database, National Institute for Health Research, University of York, Center for Reviews and Dissemination (CRD42024602349). Literature searches were performed using PubMed, Embase, and Cochrane Library databases, covering the period from their inception until 6 April 2024. The search criteria included key terms related to post-surgical rehabilitation (e.g., “physical therapy”, “postoperative rehabilitation program”), hip arthroscopy (“hip arthroscopic procedures”, “hip arthroscopy”), and femoroacetabular impingement (“FAI syndrome”, “FAIS”) (Supplementary File S1). These terms were used as keywords in titles and abstracts of retrieved articles across all databases. No time limits were applied in the literature search. Duplicates were removed, and subsequently, all records were assessed for suitability based on the abstract title and, if necessary, the full text was analyzed. Two independent authors (L.B.P., C.M.) selected the articles that met the inclusion criteria; in case of disagreement, this was resolved by the intervention of a third author (G.B.).

### 2.1. Inclusion and Exclusion Criteria

The inclusion criteria for selected studies were as follows: (1) patients who underwent hip arthroscopy for femoroacetabular impingement correction; (2) documented post-surgical rehabilitation treatment; (3) patients without previous hip pathologies or surgeries. No time or language restrictions were applied in the search.

The exclusion criteria were: (1) arthroscopic treatments for conditions other than FAI; (2) rehabilitation treatments limited to home care instructions only; (3) studies focusing exclusively on physical therapies without specifying the rehabilitation intervention; (4) studies that did not specify the type of rehabilitation intervention performed. The following study designs were excluded: meta-analyses, guidelines, conference proceedings, reviews, commentary, case report, and papers written not in English.

### 2.2. Data Extraction and Quality Assessment

Two independent authors (L.B.P., C.M.) performed data extraction from the full-text version or Supplementary Materials.

From the selected studies, the following data were extracted: general characteristics (first author, study design, and year of publication), age, type of population, surgical technique, rehabilitation treatment, main functional outcomes (patient-reported outcomes (PROs), the Hip Outcome Score for both activities of daily living (HOS-ADL) and sports activities (HOS-SS), and the modified Harris Hip Score (mHHS)), and return to sport. Due to the variability in clinical studies and the population samples analyzed, some data were either missing or could not be extracted. Consequently, these missing data were noted in the presentation of our results. The authors of the studies with missing data were not contacted.

### 2.3. Quality and Risk of Bias Evaluation

The assessment of risk of bias and quality was conducted by two independent authors (L.B.P and S.F), with any disagreements being resolved through discussion and consensus with a third author (R.A). To evaluate the included studies, the authors used the updated Risk of Bias tool (RoB 2.0) for randomized trials and the ROBINS tool for non-randomized studies, as outlined by Cochrane [12]. The overall quality of the evidence for each outcome was classified as high, moderate, low, or very low, based on the GRADE guidelines [13]. Cohen’s Kappa was used to assess inter-rater reliability in the context of risk of bias assessment using ROB 2 and ROBINS.

### 2.4. Selection of Articles

**Figure 1:**
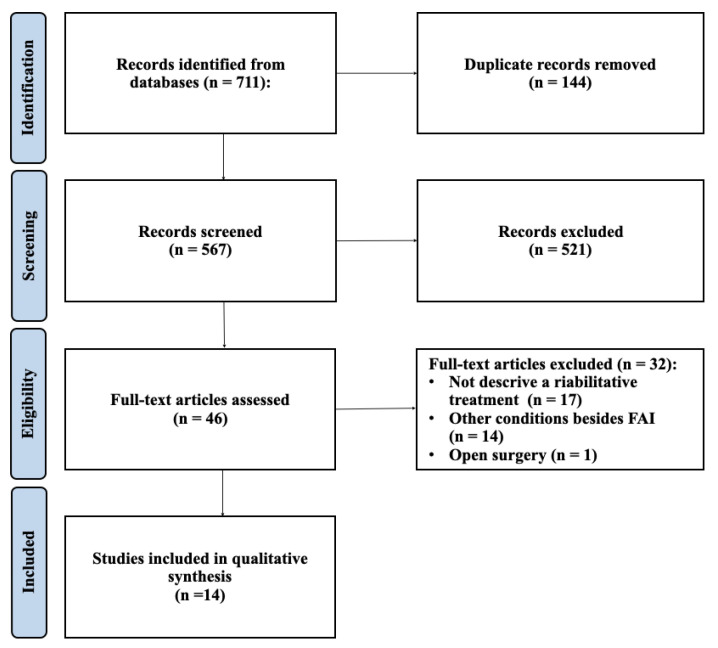
PRISMA flowchart of the article selection process.

The PRISMA flowchart for the article selection process is shown in Figure 1.

The literature search produced 432 articles selected from Pubmed, 239 articles from Embase, and 40 articles from Cochrane library. Starting from these articles, 144 duplicates were eliminated, and subsequently, 521 articles were eliminated after screening the titles and abstracts. Of the 46 remaining articles, a further 32 articles that did not fall within the inclusion criteria were eliminated; at the end of the process, 14 articles remained for the final analysis [1,2,3,5,14,15,16,17,18,19,20,21,22,23]. Of the studies analyzed, 2 studies were randomize control trial (RCT), 2 studies were case–control study, 2 studies were cohort study, 2 studies were observational study, and 6 were case series. In total, a population of 1105 patients suffering from FAI syndrome, who underwent hip arthroscopy and a postoperative rehabilitation program, were analyzed.

## 3. Results

### 3.1. Summary of Results

The treatments provided by the studies included in this review are organized into distinct phases and are based on a combination of therapeutic approaches. These include manual therapy, strengthening and balance programs, stretching, aquatic exercises, cardiovascular training, and sport-specific exercises (Table 1).

### 3.2. Manual Therapy

Manual therapy is widely used to reduce muscle tension, relieve pain, and improve hip mobility. One of the techniques mentioned in the included studies is the trigger point treatment [1,14,19]. Bennell et al. (2017) [14] described a trigger point massage technique for the rectus femoris, adductors, gluteus medius and minimus, pectineus, and tensor fascia lata muscles. This technique involves applying pressure to the point for 30 to 60 s per point, starting from the second session until the seventh session.

Additionally, soft tissue mobilization through joint and multidirectional mobilizations can be used to further enhance mobility and reduce the trigger point hypersensitivity. Dry needling may also be included as a complementary approach to treat trigger points more effectively.

Several studies have examined the efficacy of joint mobilization techniques to improve hip range of motion [1,2,3,5,18]. Cetanovich et al. (2018) [3] advocated the use of gentle passive mobilizations in all directions, including circumduction, to prevent stiffness and improve joint mobility. These mobilizations can be performed with the patient in various positions.

In addition to passive techniques, the literature describes specific mobilizations aimed at improving femoral gliding. A grade III inferior glide of the femur is applied with low speed and high amplitude at the inguinal crease, with the patient lying supine and the hip flexed at 90°, to improve flexion. Lateral-to-medial inferior glide, with the patient lying on their side and the hip abducted at 40°, is used to improve abduction, while medial-to-lateral glide improves adduction with the patient lying on the affected side. Lastly, postero-anterior glide is performed to improve external rotation and abduction, with the patient prone and the hip in a “figure-four” position.

Bennell et al. (2017) [10] and Di Benedetto et al. (2021) [19] also suggest lumbar spine mobilization when indicated by physiotherapy assessment, applying grade III or IV postero-anterior accessory glides for 30–60 s, repeated for 3–5 sets, to improve overall spinal function and, indirectly, hip mobility.

### 3.3. Aquatic Program

An aquatic rehabilitation program is introduced when clinical conditions allow. One exercise proposed by the included studies is walking in a pool, used to maintain cardiovascular fitness and improve hip range of motion [1,2,5,14,19,23]. Bennell et al. (2017) [14] recommended chest-deep water walking for 10 min in patients who underwent femoral osteochondroplasty or labral repair. For microfractures or round ligament repair, the recommended time is reduced to 5 min.

Aquatic treatment can also be used to perform exercises aimed at restoring mobility, strength, and neuromuscular control. Muller-Torrente et al. (2021) [1] proposed a series of exercises for regaining range of motion and strength, including abduction and adduction movements of the hip in chest-deep water. Another exercise involves active hip flexion and extension, with assisted flexion using a floating tube under the knee for support. Muller-Torrente et al. [1] also suggested simulated cycling movements in water, alternating between hip and knee extension and flexion.

Di Benedetto et al. (2021) [19] recommend gradually introducing strength exercises, such as mini-squats and lunges, performed in water to reduce the load on the joint.

### 3.4. Stretching

Stretching plays a crucial role in restoring full range of motion, preventing muscle contractures, and avoiding adhesions in the surgical area. Some studies recommend stretching the anterior and posterior hip capsules to improve mobility and reduce post-operative stiffness [1,14,15,19]. Bennell et al. (2017) [14] described specific techniques for stretching the anterior capsule, using a modified Thomas Test position, and the posterior capsule, with the patient lying on the unaffected side.

Several studies also highlight the importance of quadriceps stretching during rehabilitation [1,15,17,19]. Muller-Torrente et al. (2021) [1] described a standing stretch where the patient bends the knee to bring the heel towards the buttocks. Horton et al. (2021) [17] suggested a prone stretch using a strap to pull the foot towards the glutes.

In addition to these stretches, other studies recommend stretching muscles such as the piriformis, hamstrings, adductors, iliopsoas, and glutes [1,17,21]. Muller-Torrente et al. (2021) [1] proposed hamstring and adductor stretches with the patient supine, using an elastic band for support.

### 3.5. Strengthening, Core, Balance, and Proprioception

The exercise program is essential for improving hip muscle stabilization and strength. Several studies emphasize the importance of targeting the hip rotator muscles and suggest starting with isometric exercises [1,2,5,17,18,19,20,23], progressing to more dynamic movements using resistance bands and weights.

Specific exercises such as step-ups, squats, lunges, and deadlifts have been recommended, with a progression to single-leg variations [1,2,5,10,17,19,21,23]. Bennell et al. (2017) [14] suggested 3 sets of 10 repetitions at moderate-to-high intensity.

Core strengthening exercises, such as planks and bridges, are also widely recommended [1,2,19,21]. Some studies include proprioceptive exercises, such as weight-shifting and balance tasks on unstable surfaces, to improve motor control and stability [1,19,21].

### 3.6. Cardiovascular Training

Cardiovascular training is crucial for physical recovery, and studies recommend the use of stationary bicycles and elliptical machines to increase cardiovascular fitness [1,2,14,17,20,21,23]. Muller-Torrente et al. (2021) [1] recommended using a stationary bike with the saddle raised to avoid excessive hip flexion.

Frank et al. (2018) [5] introduced swimming as a cardiovascular alternative, using a pull buoy to focus on upper body movements and reduce hip stress.

### 3.7. Return to Sport

The included studies emphasize the importance of sport-specific exercises to prepare patients for competition. Plyometric and agility exercises are widely recommended to improve power, speed, and coordination [3,5,14,19,20,21]. Exercises like zig-zag running, box jumps, and agility ladder drills are commonly used.

Core training remains a key component, with advanced exercises like TRX planks and mountain climbers incorporated alongside sport-specific drills.

Different studies have reported that the percentage of return to sport is, on average, 90.3% [2,3,5,15,20,22,23].

### 3.8. Risk of Bias and Quality of Evidence

**Figure 2:**
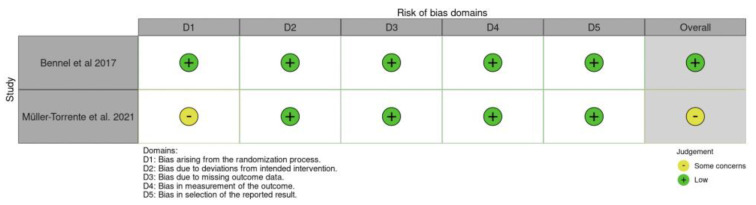
Risk of bias assessments according to RoB 2.0 tools [1,14].

**Figure 3:**
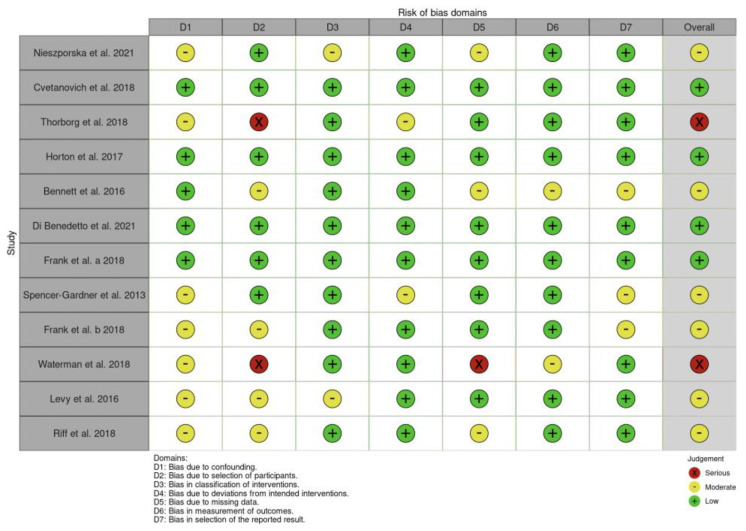
Risk of bias assessments according to the ROBINS tools [2,3,5,15,16,17,18,19,20,21,22,23,24,25].

The assessment using the RoB2 tool indicated that one paper fell into the “Some concerns” category and one in the “Low” category. The evaluation with the ROBINS-I tool revealed a diverse quality of studies, with four papers categorized as “Low”, six as “Moderate”, and two as “Serious”. Detailed results can be found in Figure 2 and Figure 3.

The authors demonstrate good agreement for ROB2 (κ = 0.71, 95% confidence interval [CI] = 0.62–0.81) and for ROBINS (κ = 0.68, 95% confidence interval [CI] = 0.59–0.77).

## 4. Discussion

The aim of this systematic review was to identify in the literature the rehabilitation protocols currently employed following hip arthroscopy, assess clinical outcomes and return-to-sport rates, and recommend an optimal evidence-based rehabilitation program. Rehabilitation after hip arthroscopy plays a crucial role in achieving successful results in treating both intra- and extra-articular hip conditions [24,25,26]. This systematic review identified that there are currently varied postoperative rehabilitation protocols for patients treated for femoroacetabular impingement with a wide range of treatment modalities, emphasizing the importance of adopting a multimodal approach in hip rehabilitation. Rehabilitation following hip arthroscopy should be individualized and based on the assessment of the individual patient, taking into account the underlying condition being treated, the type of sports activity, and functional requirements, rather than following a strictly time-based program. Although the rehabilitation program cannot be precisely divided into time-based phases, four main stages can still be identified, each characterized by specific rehabilitative elements.

The first phase is focused on joint protection and begins immediately post-surgery, aiming to promote healing and restore symmetrical range of motion. Weight-bearing is limited to 20 kg with flat-foot contact to prevent complications like femoral neck fractures [27]. Initial movements are restricted to 90° of flexion with limited rotation, and exercises like stationary cycling and isometric muscle activation are prioritized to prevent muscle atrophy and tendonitis [28].

In the second phase, patients gradually progress to full weight-bearing with exercises like mini-squats and shoulder flexion to enhance neuromuscular control and reduce compensatory movements. Soft tissue mobilization continues, targeting the prevention of scar tissue and muscle overuse [29]. Aquatic therapy is introduced to facilitate the shift to full weight-bearing [30]. Passive range of motion exercises should become more intensive, especially in rotational movements, with anterior and posterior capsule stretching initiated to help prevent adhesions and support functional flexibility.

The third phase typically focuses on restoring pre-injury function through dynamic exercises like lunges and single-leg balance drills. Treadmill walking and anti-gravity treadmill running are initiated to aid neuromuscular reeducation and cardiovascular fitness [31]. Monitoring and correcting gait and movement patterns are critical during this stage to avoid muscle overactivation and improper form.

The fourth phase of rehabilitation focuses on preparing the patient for full sports or recreational activities, emphasizing exercises that build power, endurance, and agility, such as box jumps and lateral cutting. Functional testing evaluates readiness for activities like running, jumping, and sport-specific movements without pain. Progress is monitored throughout each phase to avoid setbacks, including tendinitis or alignment issues, and ensure recovery with optimal strength and control. Since hip arthroscopy patients are often young and active, returning to sports is a key surgical outcome [32]. Returning too early may lead to recurrent pain, while an overly cautious approach could result in extended time away from sports and inadequate reconditioning before resuming full activity.

This study has some limitations, including the inclusion of studies with heterogeneous rehabilitation protocols and level of evidence, as well as the use of different scoring systems for evaluations, making it impossible to perform a quantitative assessment of the outcomes. Furthermore, it is important to highlight how the varying follow-up durations across the analyzed studies may have impacted the interpretation of the data, as well as the heterogeneity of the analyzed studies.

In conclusion, this study highlights the importance of a rehabilitation program broadly divided into four phases, where targeted exercises are tailored for each step. However, these phases are not fixed to a specific timeline but instead vary according to the patient’s individual needs, functional demands, whether they are an athlete or not, and based on the specific surgical interventions performed.

## Figures and Tables

**Table 1:** Population with femoroacetabular impingement syndrome (FAIS): Descriptive characteristics, population, age (mean ± standard deviation) surgical techniques, treatment, and main findings (protocols are not considered). HOS-ADL = Hip Outcome Score—Activities of Daily Living; HOS-SS = Hip Outcome Score—Sports-Specific; iHOT-33 = international Hip Outcome Tool; MCID = minimal clinically important difference; mHHS = modified Harris hip score; NA, not available; RCT = randomized controlled trial; ROM = range of motion; VAS = Visual Analogue Scale; THA = total hip arthroplasty; HAGOS = Copenhagen Hip and Groin Outcome Score; iHOT-12 = International Hip Outcome Tool; NAHS = Non-Arthritic Hip Score; FAA = functional activity assessment; WOCMAN = Western Ontario and McMaster Universities Osteoarthritis Index; PROs = patient-reported outcome score; LEFS = Lower Extremity Functional Scale; LSI= Limb symmetry index.

Authors	Study Design	Patients Total	Age	Population	Surgical Techniques	Treatment	Main findings
Bennel et al. 2017 [14]	RCT	30	31 ± 7 and 28.6 ± 8.1	Elite and recreational athletes	Labral repair, femoral osteochondroplasty	PT group: seven 30 min individual appointments with a study physiotherapist: one preoperative visit (after baseline assessment) within 2 weeks prior to surgery and six postoperative visits commencing at week two (approximately 2, 4, 6, 8, 10 and 12 weeks postsurgery). Control group: received no physiotherapist-prescribed rehabilitation programme	14 and 24 week post-surgery. At week 14, PT group showed significantly greater improvements on the iHOT-33 and HOS-SS (*p* < 0.05)
Müller-Torrente et al. 2021 [1]	RCT	100	41.3± 10.2 (41.8 ± 12.4, 40.9 ± 7.6)	Recreational athletes	Hip arthroscopic with an inside-out technique	PT group: physiotherapy session of 45 min each, once every two weeks for a total of 7 sessions (weeks 2, 4, 6, 8, 10, 12 and 14 post-surgery) Control Group: usual care	4 and 14 week post-surgery. The experimental group achieved statistically significant improvement over the control group in terms of: diagnostic tests, ROM, VAS (*p* < 0.001) and mHHS (*p* < 0.001) at 14 weeks
Nieszporska et al. 2021 [15]	Case-Control	12	40.1 ± 9.7	Adult patients	Standard surgery	Rehabilitation program lasting between 4 and 6 months	21.2 months. After surgery, 67% of patients returned to exercise at the same or higher level. The mean HHS results were good, with values of 88.00 ± 11.48. The SF-36 scores were >50
Cvetanovich et al 2018 [3]	Case-Control	386	33.3 ± 12.1	General population with high percentage of sports/hobbies (71.5%)	Labral repair, acetabular rim trimming, femoral osteochondroplasty, capsular repair, and acetabular delamination	4-phase rehabilitation protocol lasting between 24 and 32 weeks	2 years minimum. At minimum 2-yr follow-up, patients had statistically significant (*p* < 0.001) improvements in all PROs with a 1.2% rate of revision surgery and 1.7% rate of conversion to THA. MCID was achieved by 78.8% of patients.
Thorborg et al. 2018 [16]	Cohort Study	97 158	37.5 39	Patients undergoing hip arthroscopy	Standard 2-portal technique (anterolateral and inferior midanterior), labral reapair	4-phase rehabilitation program (mobility, stability, strength and return to sport/competition) supervised by the local physiotherapist	3, 6 and 12 months. Improvements for all HAGOS subscales and mHHS results were seen at 3 months (*p* < 0.001). Were seen only improvements for HAGOS Sport and Recreation (Sport/Rec) and Participation in Physical Activities (PA) subscales between 3 and 12 months (*p* < 0.05) but not for HAGOS Pain, Symptoms, Activities of Daily Living (ADL), or Hip-Related Quality of Life (QOL) subscales or the mHHS
Horton et al. 2021 [17]	Cohort study	51	33.94 ± 10.4 33.8 ± 10.3 33.5 ± 10.2	Patients with primary hip arthroscopy	Femoroplasty, labral repair, chondrolabaral debriment, acetabuloplasty	Intervention group: patients undergoing initial in-person visits followed by a transition to telehealth physical therapy for 3 months postoperatively. the telehealth visits consisted of up to 22 minutes of guided exercise program. Comparison group 1: patients undergoing in-person physical therapy with the same physical therapy team as the telehealth group Comparison group 2: and patients undergoing in-person therapy with a different therapy team at the same facility	3 months. There was no difference in preoperative, postoperative, or the change in iHOT-12. All groups had a significant improvement in iHOT-12 from preoperatively to the 3-month postoperative evaluation.
Bennett et al. 2016 [18]	Observational study	101	33	Active military population	-	Patients undergoing a progressive rehabilitation program by the military rehabilitation team	2, 6 and 12 months. There were significant improvements, compared with baseline, at 12-month follow-up for all measurement scales: VAS (*p* << 0.001), NHAS (*p* < 0.001) and FAA (*p* < 0.001)
Di Benedetto et al. 2021 [19]	Observational study	19	37 ± 3.8	Young adult population	Arthroscopic acetabular rim trimming and osteochondroplasty of the femoral head-neck junction	The rehabilitation process was divided into 3 phases: Phase I or protection (0 to 2 weeks p.o.)Phase II or middle (2 to 4 weeks p.o.) Phase III or advanced (4 to 6 weeks p.o.)	6 week and 3 months. Six weeks after surgery T0 and T1, there is a pain reduction of 36.04%, while at the 3-month follow-up the reduction is 33.44%. WOCMAN scale had a statistically significant improvement in activities of daily living and general performance [*p* (T0–T1) = 0.0219], *p* (T0–T2) = 0.0227]
Frank et al. 2018 [20]	Case series	58	30 ± 7.1	Recreational Athletes (cycling)	Labral repair, femoral osteochondroplasty, capsular closure, acetabular rim trimming	4-phase rehabilitation protocol: 1: protect the hip joint 2: noncompensatory gait progression 3: return to preinjury function 4: return to sport	minimum 2 years after surgery. 97% of cyclist returned to sport, on average 4.5 months after surgery. 91% ± 13% satisfaction rate in all PROs (*p* < 0.0001)
Spencer-Gardner et al. 2013 [21]	Case series	52	39.2 ± 12.2	Young adult population	-	Five-phase rehabilitation: Phase 1: day 1 to week 4 (1-2 times for week) Phase 2: weeks 4-8 (e times for week) Phase 3: weeks 8-12 (2-3 times for week) Phase 4: weeks 12-16 (1-2 times for week) Phase 5: weeks 16-24 (return to full activity/sport)	Minimum 1 year. Mean MHHS, HOS-ADL, and HOS-sport scores at a mean 12.5 (range 12–15) months were 80.1 ± 19.9 (0–100), 83.6 ± 19.2 (13.2–100), and 70.3 ± 27.0 (0–100), respectively.
Frank et al. 2018 [5]	Case series	26	31.1 ± 7.2	Recreational athletes (swimming)	Labral repair, femoral osteochondroplasty, acetabular rim trimming (T-capsulotomy)	4-phase rehabilitation protocol lasting an average of 32 weeks	2 years minimum. 100% of patients returned to swimming, on average 3.4 ± 1.7 months after surgery (54% higher level, 38% same level as before, 7% lower level). All patients showed significant improvements in PROs (*p* < 0.05).
Waterman et al. 2018 [22]	Case series	29	36 ± 11.9	Recreational athletes (golf)	Labral repair, femoral osteochondroplasty, acetabular rim trimming, capsular closure	16 to 20 weeks of a 4-phased postoperative rehabilitation program	2 years minimum. 97% of golf players returned to sports, and 55% of them noted improvement from preinjury performances.
Levy et al. 2016 [23]	Case series	51	26.3 ± 7.8	Amateur athletes (running)	Labral repair, femoral osteochondroplasty, acetabular rim trimming	4-phase rehabilitation protocol lasting 32 weeks	Minimum 2 years. 94% of patients returned to running, on average 8.5 months after surgery.Significant improvement of HOS-ADL (*p* < 0.001), HOS-SS (*p* > 0.001), and mHHS (*p* < 0.001).
Riff et al. 2018 [2]	Case series	32	34.7 ± 6.9	Amateur athletes (fitness)	Labral repair, femoral osteochondroplasty, acetabular rim trimming, and capsular closure	4-phase rehabilitation protocol lasting a mean of 32 weeks.	2 years minimum (27 +/− 6 months). 88% of patients returned to sport, and 44% of them noted improvement from preinjury performances. All patients demonstrated significant improvements in PROs (*p* < 0.05).

## Data Availability

No new data were created for this study. We really thank Alberto Basso for the statistical analysis and for the graphic design.

## Supplementary Materials

The following supporting information can be downloaded at: https://www.mdpi.com/article/10.3390/jcm13237302/s1, Supplementary File S1: Search strategy.
