# Supplementary material for: State of the Art in Rehabilitation Strategies After Hip Arthroscopy for Femoroacetabular Impingement Syndrome: A Systematic Review
Source: J Clin Med. 2024 Nov 30;13(23):7302. doi: 10.3390/jcm13237302 (PMC11642080; doi:10.3390/jcm13237302)
Supplement: Supplementary file 1 [file jcm-13-07302-s001.zip › jcm-3306093-supplementary.pdf]

	Search	Query
**PubMed search strategy**	**#1**	***Search:*** "Arthroscopy"[Mesh] AND "Femoracetabular Impingement"[Mesh] AND "Hip Joint"[MeSH]
	**#2**	***Search:*** ("hip arthroscopy"[Title/Abstract] OR "hip arthroscopy surgery"[Title/Abstract] OR "arthroscopic hip surgery"[Title/Abstract] OR "arthroscopic treatment"[Title/Abstract] OR "hip arthroscopic techniques"[Title/Abstract] OR "hip arthroscopic procedures"[Title/Abstract] OR "arthroscopic decompression"[Title/Abstract]) AND ("femoroacetabular impigment"[Title/Abstract] OR "FAI"[Title/Abstract] OR "femoroacetabular impingement syndrome"[Title/Abstract] OR "FAIS"[Title/Abstract] OR "FAI syndrome"[Title/Abstract])
	**#3**	#1 OR #2
	**#4**	***Search:*** "Rehabilitation"[Mesh] OR "Physical Therapy Modalities"[Mesh] OR "Exercise Therapy"[Mesh]
	**#5**	***Search:*** "rehabilitation"[Title/Abstract] OR "rehabilitation protocols"[Title/Abstract] OR "physical therapy"[Title/Abstract] OR "rehabilitation program"[Title/Abstract] OR "postoperative rehabilitation program"[Title/Abstract] OR "rehabilitation progression"[Title/Abstract] OR "postoperative rehabilitation protocols"[Title/Abstract] OR "postarthroscopy rehabilitation"[Title/Abstract] OR "rehabilitation targets"[Title/Abstract]
	**#6**	#4 OR #5
	**#7**	#3 AND #6
**Embase search strategy**	**#1**	***Search:*** 'hip arthroscopy'/exp AND 'femoroacetabular impingement'/exp AND 'hip'/exp
	**#2**	***Search:*** ('hip arthroscopy' OR 'hip arthroscopy surgery' OR 'arthroscopic treatment' OR 'hip arthroscopic techniques' OR 'hip arthroscopic procedures' OR 'arthroscopic decompression') AND ('femoroacetabular impigment':ti,ab OR fai:ti,ab OR 'femoroacetabular impingement syndrome':ti,ab OR fais:ti,ab OR 'fai syndrome':ti,ab)
	**#3**	#1 OR #2
	**#4**	***Search:*** rehabilitation'/exp OR 'physiotherapy'/exp OR 'kinesiotherapy'/exp
	**#5**	***Search:*** rehabilitation:ti,ab OR 'rehabilitation protocols':ti,ab OR 'physical therapy':ti,ab OR 'rehabilitation program':ti,ab OR 'postoperative rehabilitation program':ti,ab OR 'rehabilitation progression':ti,ab OR 'postoperative rehabilitation protocols':ti,ab OR 'postarthroscopy rehabilitation':ti,ab OR 'rehabilitation targets':ti,ab
	**#6**	#4 OR #5
	**#7**	#3 AND #6
**Cochrane Library search strategy**	**#1**	***Search:*** MeSH descriptor: [Arthroscopy] explode all trees
	**#2**	***Search:*** MeSH descriptor: [Femoracetabular Impingement] explode all trees
	**#3**	***Search:*** MeSH descriptor: [Hip Joint] explode all trees
	**#4**	#1 AND #2 AND #3
	**#5**	***Search:*** ("hip arthroscopy" OR "hip arthroscopy surgery" OR "arthroscopic hip surgery" OR "arthroscopic treatment" OR "hip arthroscopic techniques" OR "hip arthroscopic procedures" OR "arthroscopic decompression") AND ("femoroacetabular impigment" OR "FAI" OR "femoroacetabular impingement syndrome" OR "FAIS" OR "FAI syndrome")
	**#6**	#4 OR #5
	**#7**	***Search:*** MeSH descriptor: [Rehabilitation] explode all trees
	**#8**	***Search:*** MeSH descriptor: [Physical Therapy Modalities] explode all trees
	**#9**	***Search:*** MeSH descriptor: [Exercise Therapy] explode all trees
	**#10**	#7 OR #8 OR #9
	**#11**	***Search:*** "rehabilitation" OR "rehabilitation protocols" OR "physical therapy" OR "rehabilitation program" OR "postoperative rehabilitation program" OR "rehabilitation progression" OR "postoperative rehabilitation protocols" OR "postarthroscopy rehabilitation" OR "rehabilitation targets"
	**#12**	#10 OR #11
	**#13**	#6 AND #12

Supplementary 1. Search strategy
